# Bridging the Gap: A Quality Improvement Project Improving Communication Between Community-Based Inpatient and Outpatient Psychiatric Providers

**DOI:** 10.7759/cureus.116485

**Published:** 2026-09-18

**Authors:** Tiffany Joseph, Jonathan Zalkin, Nicole Sweat, Joseph Pullara

**Affiliations:** 1 Department of Psychiatry, Lakeland Regional Health Medical Center, Lakeland, USA

**Keywords:** community health research, hospital discharge, inpatient and outpatient setting, medical communication, system based research and organized psychiatry

## Abstract

Gaps in communication between inpatient and outpatient psychiatric providers can lead to poor care continuity, negatively impacting patient outcomes such as increased readmission rates and worsened mental health symptoms. This quality improvement project aimed to address these communication gaps by implementing a structured discharge summary communication process between inpatient psychiatric services at a large community-based academic medical center and outpatient psychiatric providers at a local clinic. A pre- and post-intervention survey design was used to assess provider perceptions regarding the timeliness, completeness, and usefulness of discharge summaries. Between January and March 2025, 210 discharge summaries were faxed from the inpatient unit to outpatient facilities. Providers at the outpatient clinic reported improvements in the timeliness of discharge summaries, with 60% expressing satisfaction with the speed of delivery. However, significant challenges remained, including insufficient medication detail and low confidence in managing post-discharge medication regimens. Despite these challenges, 100% of respondents agreed that receiving discharge summaries in advance improved patient care. The results suggest that structured discharge communication can improve continuity of care and provider confidence, but further improvements are needed in the completeness of medication details. Standardizing discharge summaries and refining the communication process could enhance patient safety, reduce readmission rates, and improve medication management post-discharge. Future efforts should focus on improving discharge documentation, addressing gaps in medication information, and extending this process to other psychiatric units and community-based healthcare settings.

## Introduction

Potential gaps in communication between inpatient and outpatient providers are especially concerning given the prevalence of serious psychiatric disorders; approximately one in five U.S. adults live with a mental illness [1], highlighting the widespread impact of poor care continuity on both individual patients and the broader healthcare system. Inpatient psychiatric hospitalization represents a critical juncture in the care of individuals with mental health conditions, yet it is often followed by significant challenges in transitioning patients to outpatient care. In a review of psychiatric inpatient records for 189 Medicaid recipients, Smith et al. [2] found that only 44% to 62% of patients attended an outpatient mental health appointment within seven days of discharge, depending on whether they had been engaged in outpatient care before admission. Research shows that poor continuity of care during this transition contributes to negative outcomes, such as higher rates of readmission, medication errors, and worsened mental health symptoms. Additionally, in a general psychiatric outpatient population rather than a specifically post-discharge cohort, non-attendance at outpatient appointments has been associated with a greater burden of disease on patient-reported and subjective outcomes, including poorer social functioning and increased symptom severity [3]. Smith et al. also found, in that same review of medical records from two inpatient psychiatric hospitals, that communication between inpatient staff and outpatient providers was associated with increased odds of attending a timely outpatient follow-up appointment after discharge, particularly among patients who had not received outpatient care in the 30 days before admission [2].

Currently, communication of discharge information between inpatient psychiatric providers at Lakeland Regional Health (LRH) and outpatient psychiatric providers at Peace River Center (PRC), both located in Florida, USA, is limited to prescribed discharge medications. Patients are mostly advised to follow up with outpatient psychiatry within three to five days of discharge. Physician-written discharge summaries, which include crucial information about the hospital course, such as medication changes, administration dates for long-acting injectables, and relevant treatment updates, are not routinely faxed upon discharge. This lack of timely communication before outpatient follow-up appointments and difficulty in the outpatient clinic identifying patient contact information have been identified as a significant barrier to effective outpatient care, as PRC largely functions as a walk-in clinic. The discharge summary has been described as the principal handoff communication document at this transition [4], and delay in its dissemination is associated with a graded increase in the risk of hospital readmission [5]. While some mitigation strategies, such as retrievable electronic documentation, have been implemented, this approach has proven insufficient in local practice because it requires the outside facility to take the additional step of requesting the record. This observation reflects the established workflow between LRH and PRC rather than a published finding.

The gap in communication remains a significant unaddressed issue, with outpatient providers at PRC reporting difficulty in tracking medication changes or obtaining pertinent contact information for patients prior to follow-up appointments. As a result, patients may not receive timely medication adjustments, which can lead to exacerbations of symptoms and unnecessary readmissions. This lack of coordination can have detrimental effects on patient safety, particularly in cases where medication changes are needed urgently after hospitalization. A systematic review of communication at hospital discharge found that a discharge summary was available at the first post-discharge visit in only 12% to 34% of cases and that this deficit adversely affected the quality of care in approximately 25% of follow-up visits [6]. In a retrospective study of 16,496 general medical admissions, Li et al. [5] found that the absence of a discharge summary was associated with a 79% increase in the rate of readmission within seven days of discharge (95% CI, 42%-124%) and a 37% increase within 28 days (95% CI, 17%-61%). The need for a reliable and efficient process for sharing discharge summaries between inpatient and outpatient providers is therefore critical to improving patient outcomes and reducing the burden of psychiatric readmissions.

This quality improvement project aims to address this critical gap in care coordination by implementing a structured discharge summary communication process. The primary purpose is to improve the timeliness and completeness of discharge information shared between community-based inpatient psychiatric services and outpatient psychiatric providers. The discharge summaries in this intervention were completed by providers on the day of patient discharge, compared to the institutional baseline delay of up to 24 hours. By enhancing the structural content of the summaries and the workflow, this project seeks to ensure that outpatient providers have the necessary discharge information to enhance provider perception and optimize communication processes.

## Materials and methods

Project design

This quality improvement project employed a pre- and post-intervention survey design adapted from real-world provider feedback to evaluate whether a structured discharge summary communication process could improve care coordination between inpatient psychiatric patients at a large community-based academic medical center in Central Florida, USA, and outpatient psychiatric providers at a single, local outpatient facility within the community. The primary aim was to enhance continuity of care by improving the timeliness and completeness of discharge information shared after inpatient psychiatric hospitalization. This quality improvement study was exempt from formal IRB review. 

Setting

The project was conducted at the inpatient psychiatric unit of a large community-based academic medical center and at a single, community-based outpatient psychiatric clinic located in Central Florida, USA. The inpatient unit provides acute stabilization to a broad psychiatric patient population, while the outpatient facility delivers follow-up care with a focus on uninsured or underinsured individuals.

Data collection

Two investigator-developed 10-question surveys (see Appendix A) were distributed using the SurveyMonkey internet platform (SurveyMonkey Inc., San Mateo, CA, USA). Each survey question consisted of 5-point Likert scale multiple-choice responses. These surveys assessed provider perceptions regarding notification of patient hospitalizations, the effectiveness of current communication workflow, confidence in medication knowledge after discharge, and perceived barriers to continuity of care. Surveys were disseminated via email with coordination from the outpatient provider manager and were completed electronically. The pre-intervention survey was distributed on January 13, 2025, and final responses were received on January 23, 2025. Eleven of 14 outpatient providers responded, and responses were submitted anonymously. Due to limited participation in the initial survey, the post-intervention survey required respondents to identify themselves by name to ensure completion tracking. The post-intervention survey was distributed on March 12, 2025, with final responses received by July 14, 2025. A total of five outpatient providers completed the post-intervention survey.

Intervention

Between January 21, 2025, and March 7, 2025, the senior inpatient behavioral health program manager faxed discharge summaries from all adult patients to the designated outpatient clinic on the business day following a patient’s discharge. All adult patients were eligible regardless of psychiatric diagnoses, without specific diagnostic exclusion or inclusion criteria. The referral site was determined by patient insurance status, as outpatient psychiatric services only accept specific insurance plans. During the intervention period, 210 discharge summaries were successfully transmitted from the inpatient unit to outpatient facilities. Of those, only eight were faxed to internal network outpatient providers, with the remainder sent to the outside partner community-based clinic. Because so few patients were routed to internal network outpatient clinics for follow-up, these providers did not meaningfully participate in the intervention and were therefore excluded from the post-intervention analysis. The faxed discharge summaries included updated medication lists, recent long-acting injectable administration dates, and clinical details necessary for outpatient treatment planning.

Evaluation of measures

Provider perceptions regarding timeliness, accuracy, and usefulness of discharge summaries were compared before and after the intervention. Outcomes of interest included provider satisfaction with communication workflow, provider confidence in managing medication regimens after hospitalization, and perceived impact on continuity of patient care. Due to the anonymity of pre-intervention responses, individual respondents could not be matched between survey phases. Therefore, analysis is limited to descriptive statistics, including frequency counts and percentage distributions of responses from the two independent groups.

## Results

Intervention reach and results

During the intervention period from January 21, 2025, through March 7, 2025, the inpatient behavioral health unit faxed 210 discharge summaries to outpatient psychiatric facilities. Of these transmissions, 202 (96%) were faxed to the partner clinic for insurance-directed follow-up referrals, while only eight (4%) discharge summaries were sent to the hospital’s internal network outpatient psychiatry offices. Because so few patients were eligible for follow-up at the internal outpatient clinics, these providers did not meaningfully engage with the intervention, limiting post-intervention evaluation to partner providers. A total of 11 outpatient providers completed the pre-intervention survey, while five partner providers completed the post-intervention survey. Because pre-intervention surveys were anonymous and respondents could not be matched to post-intervention responses, analysis was restricted to descriptive statistics comparing grouped response trends.

Pre-intervention provider perceptions

Before the intervention, a significant portion of respondents expressed concerns about the communication process between inpatient and outpatient providers. A majority of providers (8/11, 73%) reported that they were always or usually notified about patient hospitalizations. However, confidence in the current handoff process was low, with 8/11 respondents (73%) indicating they were either not confident or neutral about the current system. The effectiveness of the existing communication methods, such as discharge summaries, was rated as 'somewhat effective' by 6/11 respondents (55%), with a substantial portion also deeming them 'somewhat ineffective' (Table 1). 

**Table 1:** Pre-intervention survey responses Note: Total n = 11. The response distribution column reports the number of respondents selecting each option; options that received no responses are omitted. The key finding column gives the summary value referred to in the text. Every respondent answered every item.

Survey topic	Response distribution (total n = 11)	Key finding
Notification of hospitalization	Always = 4, usually = 4, rarely =2, never = 1	Always or usually = 8/11 (73%)
Confidence in current handoff	Somewhat confident = 3, neutral = 4, not so confident = 3, not at all confident = 1	Not confident or neutral = 8/11 (73%)
Effectiveness of communication methods	Extremely effective = 1, somewhat effective = 6, somewhat ineffective = 3, not effective at all = 1	Somewhat effective = 6/11 (55%)
Usefulness of more detailed discharge summaries	Extremely useful = 7, somewhat useful = 3, neutral = 1	Extremely useful = 7/11 (64%)
Timeliness of discharge summaries as a barrier	Extremely significant = 4, somewhat significant = 5, neutral = 1, somewhat insignificant = 1	Somewhat or extremely significant = 9/11 (82%)
Patient non-adherence to follow-up as a barrier	Extremely significant = 5, somewhat significant = 4, neutral = 1, somewhat insignificant = 1	Somewhat or extremely significant = 9/11 (82%)
More comprehensive discharge summaries would improve handoffs	Extremely helpful improvement = 1, significant improvement = 5, moderate improvement = 4, slight improvement = 1	Significant or extremely helpful improvement = 6/11 (55%)
Standardization would improve handoffs	Extremely helpful improvement = 2, significant improvement = 6, moderate improvement = 3	Significant or extremely helpful improvement = 8/11 (73%)
Overall satisfaction with handoff	Somewhat satisfied = 2, neutral = 4, somewhat dissatisfied = 4, very dissatisfied = 1	Dissatisfied or neutral = 9/11 (82%)

There was a clear demand for more detailed discharge summaries, as 7/11 respondents (64%) found them to be extremely useful. Timeliness emerged as a significant barrier, with 9/11 respondents (82%) indicating that delayed discharge summaries were a major challenge. Patient non-adherence to follow-up was also identified as a barrier to effective handoff, with 9/11 respondents (82%) considering it somewhat or extremely significant. Overall, 6/11 respondents (55%) indicated that more comprehensive discharge summaries and documentation would be a significant or extremely helpful improvement, with a further 4/11 (36%) rating it a moderate improvement, and 8/11 (73%) rated standardizing handoff procedures a significant or extremely helpful improvement. Nine of 11 providers (82%) expressed dissatisfaction or neutrality with the current system.

The pre-intervention survey results revealed that providers identified several key barriers to effective communication between inpatient and outpatient psychiatric teams. Among the most frequently reported barriers was the timeliness of discharge summaries, with nine out of 11 providers (82%) considering delays in receiving discharge summaries as either somewhat or extremely significant. Additionally, patient non-adherence to follow-up was also a major concern, with nine out of 11 providers (82%) deeming it either somewhat or extremely significant. Incomplete or missing discharge summaries were the third most frequently identified barrier, with eight out of 11 providers (73%) highlighting it as a significant issue (Figure 1). These findings underscore the critical role that timely and complete discharge summaries play in facilitating smooth transitions of care and emphasize the need for improving the handoff process to address these challenges.ima

**Figure 1:**
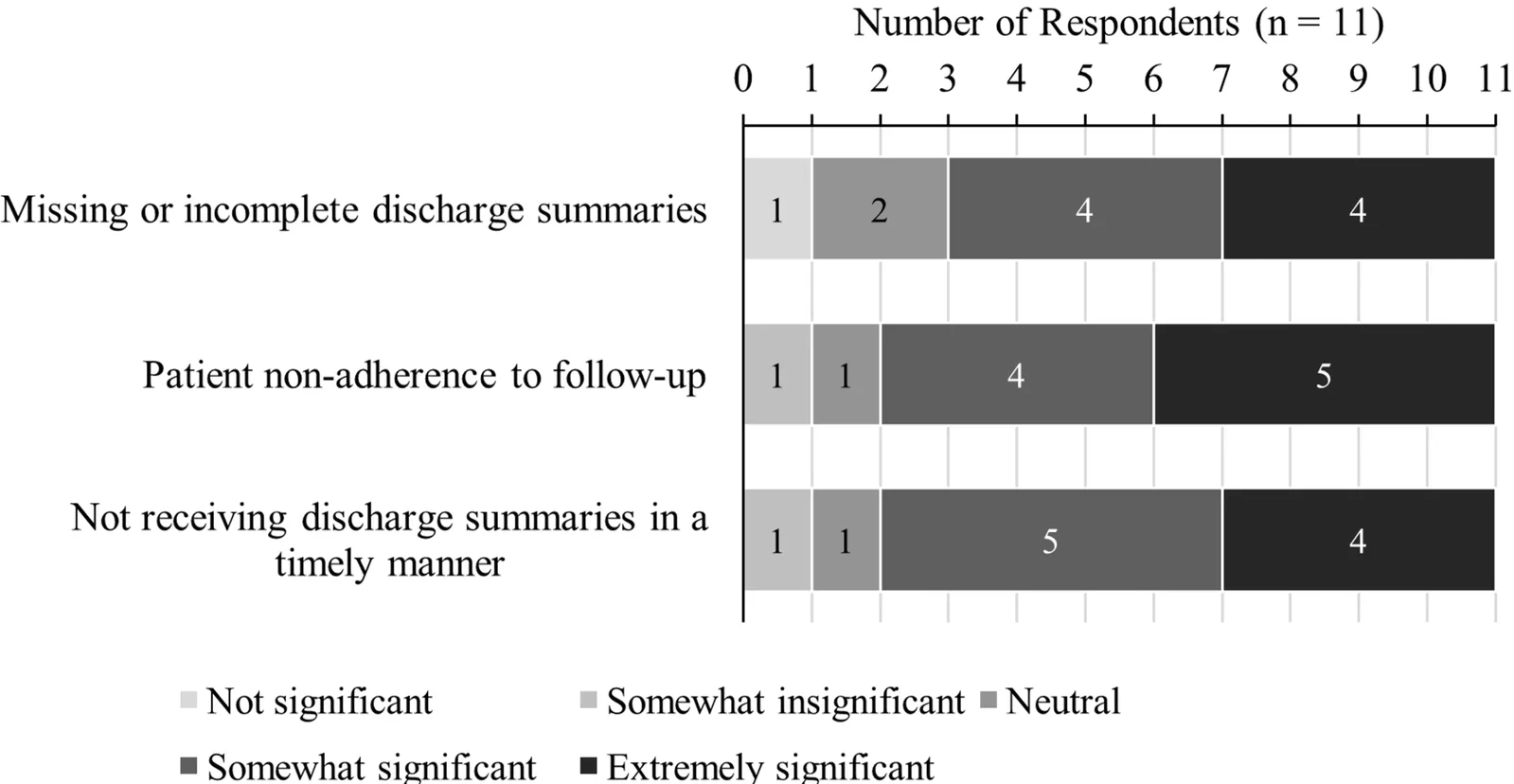
Provider-perceived barriers to effective inpatient-to-outpatient psychiatric handoff before intervention Note: Total n =11. Segments show the full response distribution for each barrier across the five-point significance scale; segments with no responses are not labeled. Combining the two highest categories, the proportion rating each item as somewhat or extremely significant was 9/11 (82%) for untimely discharge summaries, 9/11 (82%) for patient non-adherence to follow-up, and 8/11 (73%) for missing or incomplete discharge summaries.

Post-intervention provider perceptions

After the intervention, PRC providers reported mixed results across communication and care management. Two of five respondents (40%) were satisfied with the timeliness of receiving discharge summaries, one (20%) was neutral, and two (40%) were dissatisfied or very dissatisfied. Confidence in medication knowledge remained a challenge, with 3/5 respondents (60%) reporting that they were not so confident or not confident at all in their understanding of medication changes post-discharge (Table 2).

Despite these concerns, all five respondents (5/5, 100%) agreed or strongly agreed that receiving discharge summaries before follow-up appointments improved their ability to manage patient care. The completeness of discharge summaries, however, remained a concern, with only 2/5 providers (40%) reporting that summaries were usually complete and accurate, 2/5 (40%) reporting that they were sometimes complete, and 1/5 (20%) reporting that they were never complete. Separately, 4/5 providers (80%) rated the detail provided on medication adjustments as insufficient or not at all sufficient. When asked about the impact on medication errors, 3/5 providers (60%) agreed or strongly agreed that the intervention reduced the risk of errors. Communication with inpatient providers showed little change, with only 2/5 respondents (40%) reporting that communication had somewhat improved and 3/5 (60%) reporting no change at all. Access to discharge summaries was rated easy by 2/5 respondents (40%), neutral by 1/5 (20%), and difficult or very difficult by 2/5 (40%), though this still left some providers struggling with timely access. Finally, when asked if they would recommend continuing the faxing process, 3/5 respondents (60%) were likely or very likely to endorse continuing the intervention, while 1/5 (20%) was neutral and 1/5 (20%) was very unlikely to do so (see Table 2).

**Table 2:** Post-intervention survey responses Note: Total n = 5. The response distribution column reports the number of respondents selecting each option on the 5-point Likert scale used for that item; options that received no responses are omitted. The key finding column gives the summary value referred to in the text. Every respondent answered every item. Because the denominator is five, each respondent represents 20% of the sample, and small differences between items should not be overinterpreted.

Survey topic	Response distribution (total n = 5)	Key finding
Timeliness of discharge summaries	Satisfied = 2, neutral = 1, dissatisfied = 1, very dissatisfied = 1	Satisfied = 2/5 (40%)
Confidence in medication knowledge	Confident = 2, not so confident = 2, not confident at all = 1	Not confident = 3/5 (60%)
Receiving summaries before follow-up improves care	Strongly agree = 4, agree = 1	Agree or strongly agree = 5/5 (100%)
Completeness of discharge summaries	Usually = 2, sometimes = 2, never = 1	Usually complete = 2/5 (40%)
Perceived reduction in medication errors	Strongly agree = 1, agree = 2, neutral = 1, disagree = 1	Agree or strongly agree = 3/5 (60%)
Communication with inpatient providers	No change = 3, somewhat improved = 2	Somewhat improved = 2/5 (40%)
Ease of access to summaries	Easy = 2, neutral = 1, difficult = 1, very difficult = 1	Easy = 2/5 (40%)
Adequate detail on medication adjustments	Somewhat sufficient = 1, somewhat insufficient = 3, not at all sufficient = 1	Insufficient or not at all sufficient = 4/5 (80%)
Recommend continuation of process	Very likely = 2, likely = 1, neutral = 1, very unlikely = 1	Likely or very likely = 3/5 (60%)

The post-intervention survey results indicate that the intervention led to positive changes in several key areas, though some challenges persist. All five providers (5/5, 100%) agreed or strongly agreed that receiving discharge summaries before follow-up appointments significantly improved their ability to manage patient care, highlighting the intervention's positive impact on clinical decision-making. In terms of medication safety, 3/5 providers (60%) believed that the intervention helped reduce medication errors. Additionally, only 2/5 providers (40%) felt that communication with inpatient providers had somewhat improved, while 3/5 (60%) reported no change (Figure 2). These are provider perceptions only; no objective measures of medication errors, readmission, or other patient outcomes were collected. They should also be read alongside the unresolved concerns reported by the same five respondents, of whom 4/5 (80%) rated the medication-adjustment detail in discharge summaries as insufficient, and 3/5 (60%) remained not confident in their knowledge of post-discharge medication changes. These results suggest that while the intervention improved some aspects of care, further refinement in the discharge summary content and the timeliness of communication is necessary to fully address providers’ concerns.

**Figure 2:**
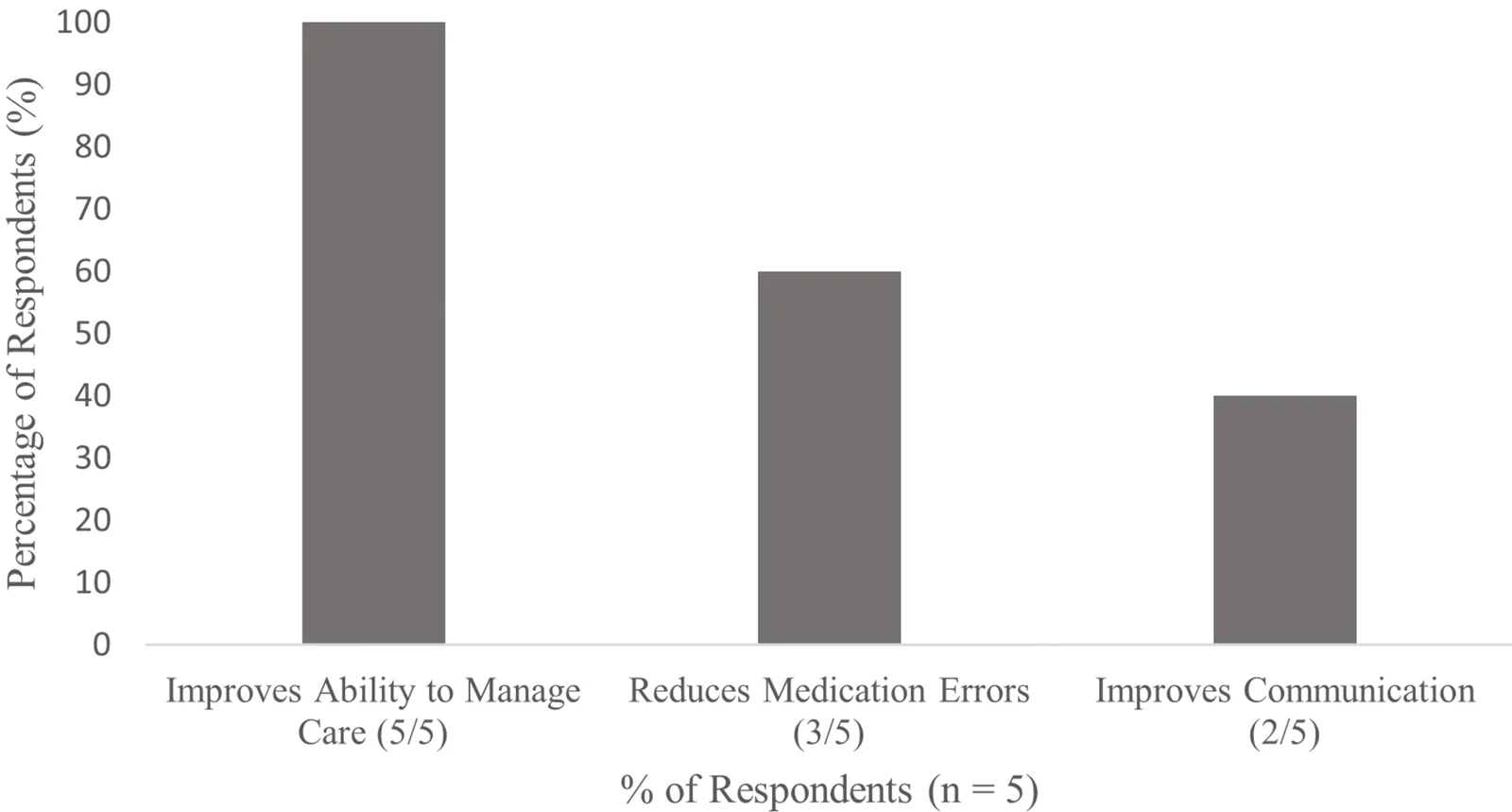
Provider-perceived changes following the intervention (post-intervention respondents only) Note: Total n = 5. Bars show the number and proportion of post-intervention respondents giving a positive response to three items: whether receiving discharge summaries before follow-up appointments improved their ability to manage patient care (agree or strongly agree = 5/5, 100%); whether the intervention reduced the risk of medication errors (agree or strongly agree = 3/5, 60%); and whether communication with inpatient providers had improved (somewhat improved = 2/5, 40%, with the remaining 3/5, 60%, reporting no change).

Pre- vs. post-intervention comparisons

A descriptive comparison of pre- and post-intervention responses is presented below. Because the two surveys were completed by independent, non-matched groups of respondents and did not use identical items, these comparisons describe grouped provider perceptions and do not represent within-person change. Timeliness of discharge summaries remained a concern in both phases, though there was a slight improvement in the post-intervention phase. The most notable improvement was in providers' perceptions of care management, with 5/5 post-intervention respondents (100%) agreeing that receiving discharge summaries before follow-up improved their ability to manage patient care. By comparison, 7/11 pre-intervention respondents (64%) rated more detailed discharge summaries as extremely useful; the two items were worded differently and are not directly comparable. Communication with inpatient providers showed little change, with only 2/5 post-intervention respondents (40%) reporting improvement and 3/5 (60%) reporting none, and confidence in medication knowledge did not improve meaningfully, remaining low in both phases (Table 3). The general trend suggests that while the intervention led to some improvements, significant challenges related to medication detail and overall timeliness remain. While survey results suggest improvement in some provider-perceived domains following the intervention, most respondents reported no change in communication with the inpatient team, confidence in medication changes did not improve, and continued concerns about inadequate medication detail highlight the need for further strengthening of discharge documentation.

**Table 3:** Descriptive comparison of survey domains Note: Pre-intervention total n = 11; post-intervention total n = 5. The pre- and post-intervention surveys were completed by two independent groups of respondents who could not be matched, and the survey items were not identical across the two phases. This table is therefore a descriptive comparison of grouped provider perceptions and does not represent within-person change or a statistically testable pre/post difference. Values are the number of respondents endorsing the listed categories out of the total responding, with the corresponding percentage.

Domain	Pre-intervention survey (total n = 11)	Post-intervention survey (total n = 5)	Descriptive comparison
Timeliness of discharge summaries	Delay rated as a somewhat or extremely significant barrier = 9/11 (82%)	Satisfied = 2/5 (40%); neutral = 1/5 (20%); dissatisfied or very dissatisfied = 2/5 (40%)	Different items were used in each phase, and the groups were not matched; no inferential comparison is possible.
Confidence in medication changes	Not confident or neutral about the current handoff process = 8/11 (73%)	Not confident in knowledge of post-discharge medication changes = 3/5 (60%)	Confidence remained low after the intervention; no meaningful improvement was demonstrated. This is a key unresolved finding.
Perceived communication effectiveness	Existing communication methods rated somewhat effective = 6/11 (55%)	Somewhat improved = 2/5 (40%); no change = 3/5 (60%)	Different items were used in each phase, and the groups were not matched; no inferential comparison is possible.
Medication-related concerns	Patient non-adherence to follow-up rated a somewhat or extremely significant barrier = 9/11 (82%)	Agree or strongly agree that the intervention reduced medication-error risk = 3/5 (60%); medication-adjustment detail still rated insufficient = 4/5 (80%)	Different constructs were measured in each phase. The persistent 80% insufficiency rating indicates an unresolved problem rather than a benefit.

Summary

The intervention increased the timely transfer of discharge summaries to outpatient providers, and the five PRC providers who completed the post-intervention survey perceived it as beneficial to patient care. Providers agreed that receiving discharge information in advance improves their ability to manage clinical needs; however, limitations in medication detail and low confidence in medication knowledge remained significant and largely unchanged challenges, with 4/5 respondents (80%) rating the medication-adjustment detail as insufficient and 3/5 (60%) remaining not confident in their knowledge of post-discharge medication changes. These findings underscore the importance of refining discharge documentation and strengthening communication workflows to sustain and expand improvements in continuity of care.

## Discussion

This quality improvement project sought to improve the timeliness and completeness of discharge information shared between inpatient psychiatric providers at LRH and outpatient psychiatric providers at PRC to enhance continuity of care. The findings suggest that the implementation of a structured discharge summary communication process was associated with improved provider-reported perceptions of care coordination, although most respondents reported no change in day-to-day communication with the inpatient team, which is consistent with research showing the importance of timely and accurate discharge summaries in preventing readmissions and medication errors. Previous studies have highlighted the critical role of communication between inpatient and outpatient providers, particularly for patients with psychiatric conditions. Improved relational continuity of care for patients with serious mental illness could help reduce premature deaths and suicides, decrease emergency department visits, and enhance overall quality of life [7].

The pre- and post-intervention survey results from this project indicated that providers at PRC experienced some improvements in their ability to manage care when discharge summaries were received before follow-up appointments. As seen in the post-intervention survey, all five respondents (100%) agreed that receiving discharge summaries in advance improved patient care, although this unanimity rests on a denominator of only five providers. These findings align with those of Smith et al. [2], who demonstrated that better communication between inpatient and outpatient providers increased the likelihood of patients attending outpatient follow-up appointments after discharge. Their study also highlighted that communication with outpatient providers may be especially important for patients who were not engaged in outpatient care prior to admission [2]. This improvement could be attributed to the enhanced communication process established by the intervention, which facilitated better-informed care decisions for patients post-discharge.

Despite the positive trends, the results also revealed persistent challenges in the areas of medication detail and provider confidence in managing post-discharge medication regimens. Most PRC providers (4/5, 80%) felt that the discharge summaries provided insufficient detail on medication changes, and 3/5 providers (60%) were not confident in their medication knowledge after receiving the summaries. Taken together, these two findings represent the most important unresolved problem identified by this project: the intervention improved the transmission of discharge summaries without demonstrably improving the adequacy of their medication content. This lack of confidence in medication adjustments is particularly concerning given the heightened risks associated with psychiatric medication management following discharge. This leads to further consideration of creating standardization amongst the physician-written summaries to ensure completeness [8].

A study by Stockdale et al. [9] found that organizational barriers, such as a lack of financial resources, a lack of integrated information technology, and organizational cultures not conducive to change, hinder communication between inpatient and outpatient mental health clinicians. Other work has demonstrated that the content quality of discharge summaries improves following a structured curriculum and formal teaching for the clinicians who write them [10]. Standardized, template-driven discharge summaries embedded in the electronic health record have likewise been implemented at the institutional level, with improved content completeness and greater satisfaction among the providers receiving them [11]. Thus, further work is needed to ensure that discharge summaries include comprehensive and actionable information on medication adjustments, especially for patients who may require complex pharmacological interventions [12,13].

Additionally, the intervention improved the timeliness with which discharge summaries reached PRC, although only 2/5 providers (40%) reported being satisfied with the speed of delivery, reinforcing the notion that timely communication is essential for successful patient transitions from inpatient to outpatient care. However, further efforts should be made to standardize and refine the discharge documentation. In this study, although discharge summaries were transmitted promptly, gaps in the details regarding medication adjustments may still pose a risk to patient safety and reduce the effectiveness of the intervention, since unintended medication discrepancies at discharge are a well-described source of post-discharge adverse drug events [12,13]. It is essential to ensure that all relevant clinical information is conveyed in a clear, structured format to empower outpatient providers to make informed treatment decisions.

Limitations

There are several limitations to this quality improvement project that should be considered when interpreting the results. First, patient insurance status significantly influenced outpatient referral patterns, resulting in the exclusion of LRH outpatient psychiatric providers from the post-intervention survey analysis due to insufficient patient volume. Discharged patients were referred for follow-up based on insurance coverage, and because LRH outpatient psychiatry accepts only specific insurance plans, the vast majority of referrals were directed to PRC instead. As a result, most LRH outpatient providers did not directly experience the communication intervention and therefore could not meaningfully evaluate its impact.

Additionally, the pre-intervention survey was conducted anonymously, preventing the ability to separate LRH provider responses from PRC provider responses and limiting the internal validity of the baseline data. Anonymity also precluded respondent-level comparison of pre- and post-intervention responses, reducing the ability to assess whether changes in satisfaction or confidence occurred at the individual level. The change of anonymity may have impacted response patterns and provider satisfaction with the intervention.

Several challenges existed with survey design and provider engagement. The surveys were not piloted, nor were content validity or reliability assessed. The survey questions were varied in structure rather than standardized using validated scaling measures, restricting the ability to conduct inferential statistical analysis. Due to these limitations, results were analyzed using descriptive statistics only. Provider participation also impacted the robustness of findings. Only 11 of 14 eligible providers completed the pre-intervention survey. Follow-up participation also initially remained low, with only two of five PRC providers completing the post-intervention survey within the first month. Multiple reminders were required to secure responses, raising the possibility that survey fatigue or frustration may have contributed to negatively skewed feedback. The post-intervention denominator of five providers also means that each respondent represents 20% of the sample, so a change in a single response produces a large apparent shift in the reported percentages and may lead to response bias.

Lastly, resource limitations also affected survey quality. The free version of the SurveyMonkey platform restricted surveys to 10 multiple-choice questions, eliminating the opportunity to include open-ended questions that could have provided richer qualitative insights into communication challenges or suggestions for workflow improvement. Also, the study evaluated provider perceptions only; objective clinical outcomes such as documented medication-related errors, readmission rates, or follow-up adherence were not measured. The relatively short intervention period further limited the ability to assess sustained improvements in communication processes or patient safety outcomes. Several survey items also combined qualitatively different Likert categories, such as 'satisfied or neutral' and 'sometimes or usually complete,' which limits the interpretability of those results and may overstate the proportion of favorable responses. These limitations collectively reduce generalizability and constrain the strength of conclusions that can be drawn regarding intervention effectiveness. Future efforts should incorporate validated measurement tools, improved participant engagement strategies, and objective outcome data over a longer time frame to better assess the impact of communication enhancements on continuity of psychiatric care.

## Conclusions

This quality improvement project suggests that implementing a structured discharge summary communication process between inpatient and outpatient psychiatric providers can improve provider-perceived care coordination. Perceived communication with the inpatient team did not improve for most respondents, and no objective clinical outcomes were measured, so the effect on patient care remains unknown. Providers at PRC reported benefits from receiving discharge summaries in advance, particularly for continuity of care and clinical decision-making. Confidence in post-discharge medication changes did not improve, however, and most post-intervention respondents continued to rate the medication-adjustment detail in discharge summaries as insufficient. The intervention therefore improved the transmission of discharge summaries without demonstrably improving their medication content. However, challenges remain regarding the completeness of medication information, which must be addressed to fully realize the potential benefits of improved discharge communication. Future efforts should focus on refining discharge documentation, ensuring that detailed medication changes are included, and exploring additional methods to enhance provider confidence in managing post-discharge care. Expanding this process to other psychiatric units and healthcare settings, while addressing the gaps identified in this study, could detail a methodology that can be applied to broader systems.

## Appendices

Appendix A

Pre-Intervention Survey

Section 1: Confidence in handoff process

1. Of your patients who become hospitalized, how often are you notified?

A.      Always

B.      Usually (75% of the time)

C.      Sometimes (50% of the time)

D.      Rarely (25% of the time)

E.      Never

2. How confident are you in the current handoff process between inpatient and outpatient providers?

A.      Extremely confident

B.      Somewhat confident

C.      Neutral

D.      Not so confident

E.      Not at all confident

3. How effective do you find the current methods of communication (e.g., discharge summaries, referral notes)?

A.      Extremely effective

B.      Somewhat effective

C.      Neutral

D.      Somewhat ineffective

E.      Not effective at all

Section 2: Communication and coordination

How useful do you think the following communications would be in improving the handoff process?

4. More detailed discharge summaries

A.      Extremely useful

B.      Somewhat useful

C.      Neutral

D.      Not so useful

E.      Not at all useful

Section 3: Barriers to effective handoff

How significant do you think the following barriers are to effective handoff between inpatient and outpatient providers?

5. Missing or incomplete discharge summaries

A.      Not significant at all

B.      Somewhat insignificant

C.      Neutral

D.      Somewhat significant

E.      Extremely significant

6. Not receiving discharge summaries in a timely manner

A.      Not significant at all

B.      Somewhat insignificant

C.      Neutral

D.      Somewhat significant

E.      Extremely significant

7. Patient non-adherence to follow-up

A.      Not significant at all

B.      Somewhat insignificant

C.      Neutral

D.      Somewhat significant

E.      Extremely significant

Section 4: Suggestions for improvement

How much improvement would you expect from the following changes to the handoff process?

8. More comprehensive discharge summaries and documentation

A.      No improvement

B.      Slight improvement

C.      Moderate improvement

D.      Significant improvement

E.      Extremely helpful improvement

9. Standardization of handoff procedures

A.      No improvement

B.      Slight improvement

C.      Moderate improvement

D.      Significant improvement

E.      Extremely helpful improvement

10. Overall, how satisfied are you with the current handoff process between inpatient and outpatient providers?

A.      Very satisfied

B.      Somewhat satisfied

C.      Neutral

D.      Somewhat dissatisfied

E.      Very dissatisfied

Post-Intervention Survey

1. Please type your full name.

2. How satisfied are you with the timeliness of receiving discharge summaries for your patients after inpatient psychiatric hospitalizations?

A.      Very satisfied

B.      Satisfied

C.      Neither satisfied nor dissatisfied

D.      Dissatisfied

E.      Very dissatisfied

3. How confident are you in your knowledge of your patients' medication changes following their inpatient psychiatric hospitalization?

A.      Very confident

B.      Confident

C.      Neither confident nor unconfident

D.      Not so confident

E.      Not at all confident

4. Do you feel that receiving discharge summaries prior to follow-up appointments improves your ability to manage patient care?

A.      Strongly agree

B.      Agree

C.      Neither agree nor disagree

D.      Disagree

E.      Strongly disagree

5. How often do you feel that you receive complete and accurate discharge summaries for your patients after inpatient psychiatric stays?

A.      Always

B.      Usually

C.      Sometimes

D.      Rarely

E.      Never

6. Do you believe that the current process for receiving discharge summaries has reduced the risk of medication errors in your patients?

A.      Strongly agree

B.      Agree

C.      Neither agree nor disagree

D.      Disagree

E.      Strongly disagree

7. In your opinion, how has the availability of discharge summaries impacted your communication with inpatient providers?

A.      Significantly worsened communication

B.      Somewhat worsened communication

C.      No change

D.      Somewhat improved communication

E.      Significantly improved communication

8. How easy has it been for you to access discharge summaries in a timely manner?

A.      Very easy

B.      Easy

C.      Neither easy nor difficult

D.      Difficult

E.      Very difficult

9. Do you feel that the discharge summaries provide sufficient detail about medication adjustments and clinical recommendations?

A.      Not at all sufficient

B.      Somewhat insufficient

C.      Neither sufficient nor insufficient

D.      Somewhat sufficient

E.       Very sufficient

10. How likely are you to recommend the continuation of this discharge summary process (faxing summaries the day after discharge) to improve communication with inpatient providers?

A.      Very unlikely

B.      Unlikely

C.      Neither likely nor unlikely

D.      Likely

E.      Very likely
